# Structural and functional brain biomarkers of clinical response to rTMS of medication-resistant auditory hallucinations in schizophrenia patients: study protocol for a randomized sham-controlled double-blind clinical trial

**DOI:** 10.1186/s13063-019-3311-x

**Published:** 2019-04-23

**Authors:** Fanny Thomas, Noomane Bouaziz, Cécile Gallea, Palmyre Schenin-King Andrianisaina, Florence Durand, Ombline Bolloré, René Benadhira, Clémence Isaac, Sonia Braha-Zeitoun, Virginie Moulier, Antoni Valero-Cabré, Dominique Januel

**Affiliations:** 1 Unité de Recherche Clinique, Établissement Public de Santé Ville-Evrard, 202 avenue Jean Jaurès, 93332 Neuilly-sur-Marne, France; 2 Cerebral Dynamics, Plasticity and Rehabilitation Group, Frontlab, Centre de Recherche de l’Institut du Cerveau et de la Moelle Épinière, CNRS UMR 7225, INSERM UMRS 1127 and Université Pierre et Marie Curie, 47 boulevard de l’Hôpital, 75013 Paris, France; 3 Laboratory for Cerebral Dynamics Plasticity and Rehabilitation, Boston University School of Medicine, 700 Albany Street, Boston, MA W-702A USA; 4 Movement Investigations and Therapeutics, MOV’IT, Centre de Recherche de l’Institut du Cerveau et de la Moelle Épinière, CNRS UMR 7225, INSERM UMRS 1127 and Université Pierre et Marie Curie, 47 boulevard de l’Hôpital, 75013 Paris, France; 5 Laboratoire de psychopathologie et de neuropsychologie, Université Paris 8, 2 rue de la Liberté, 93526 Saint-Denis, France

**Keywords:** Schizophrenia, auditory verbal hallucinations, non-invasive brain stimulation, repetitive transcranial magnetic stimulation, clinical response, predictive biomarkers, structural connectivity, functional connectivity, clinical trial

## Abstract

**Background:**

The potential of non-invasive repetitive transcranial magnetic stimulation (rTMS) to improve auditory verbal hallucinations (AVH) in schizophrenia patients has been increasingly explored over the past decade. Despite highly promising results, high inter-individual variability of clinical response and ineffective outcomes in a significant number of patients underscored the need to identify factors associated with the clinical response to rTMS. It should help improve the efficacy of rTMS in patients with medication-resistant AVH, and allow a better understanding of its neural impact. Here, we describe an exploratory study protocol which aims to identify structural and functional brain biomarkers associated with clinical response after an rTMS treatment for medication-resistant AVH in schizophrenia.

**Methods:**

Forty-five schizophrenia patients with medication-resistant AVH will be enrolled in a double-blind randomized sham-controlled monocentric clinical trial. Patients will be assigned to a regime of 20 sessions of active or sham 1 Hz rTMS delivered twice a day, 5 days a week for 2 weeks over the left temporo-parietal junction. Response will be assessed after rTMS and patients will be classified in responders or non-responders to treatment. Magnetic resonance imaging (MRI) sessions including diffusion weighted imaging and resting-state functional MRI sequences will be recorded before the onset of the rTMS treatment and 3 days following its discontinuation. The primary outcome measure is difference in fractional anisotropy between responder and non-responder patients at baseline. Differences in resting-state functional MRI data at baseline will be also investigated between responder and non-responder groups. Clinical, neuropsychological, neurophysiological, and blood serum BDNF assessments will be performed at baseline, 3 days, 1 month, and 3 months following rTMS.

**Discussion:**

The aim of this research project is to identify and assess the biomarker value of MRI-based structural and functional biomarkers predicting clinical response to rTMS for AVH in schizophrenia patients. The outcome of the trial should improve patient care by offering them a novel suitable therapy and deepen our understanding on how rTMS may impact AVH and develop more effective therapies adapted to individual patient needs.

**Trial registration:**

ClinicalTrials.gov, NCT02755623. Registered on 22 April 2016.

**Electronic supplementary material:**

The online version of this article (10.1186/s13063-019-3311-x) contains supplementary material, which is available to authorized users.

## Background

Schizophrenia has been conceptualized as a brain connectivity disorder, which would be the cause of the main symptoms of the pathology [1, 2]. Auditory verbal hallucinations (AVH) are the hallmark symptom of schizophrenia, present in 70–80% of patients [3, 4] and have been linked to alterations in structural and/or functional interactions. In the structural domain, alterations of white matter integrity in the left arcuate fasciculus and interhemispheric projections via the corpus callosum have been associated with AVH in schizophrenia patients [5, 6]. Additionally, in the functional domain, deficits of resting-state functional connectivity have been reported for left fronto-temporal interactions, across nodes of the Default Mode Network (DMN) and also between DMN and Salience Network regions, and have been associated with AVH severity [6–10]. In line with the concept of altered connectivity, treatments with the ability to modulate local brain activity and network functional connectivity could serve to normalize dysfunction in perturbed networks, improving AVH severity.

Repetitive transcranial magnetic stimulation (rTMS) is a non-invasive brain stimulation technology with the ability to modulate the activity of directly targeted cortical areas and their associated networks [11]. Widely used over the last two decades in exploratory, diagnostic, and therapeutic applications, rTMS has shown a promising potential for restoring abnormal functional connectivity patterns underlying pathology. Several meta-analyses have demonstrated that low-frequency rTMS patterns delivered to the left temporoparietal junction (TPJ) significantly reduced AVH in schizophrenia [12–19]. However, the clinical benefit of this therapy remains moderate and is characterized by a high degree of interindividual variability. Indeed, a large number of patients appear to respond poorly, exhibiting no significant improvement in AVH rates [16].

Variability of clinical response to rTMS has been explained by brain connectivity differences subtending functional [20] and/or structural [21–23] interactions. Nonetheless, to date, very few studies have aimed to further characterize the detailed determinants of this variability. A better understanding of this phenomena bears the potential to increase the effectiveness of rTMS-based treatments and promote the development of individualized therapeutic approaches tailored to the specificities of each patient. The identification of predictive and explanatory biomarkers of clinical response to rTMS is an expanding domain with the potential to improve therapeutic plans offered to patients, while improving our understanding of underlying rTMS mechanisms on pathological symptoms such as AVH. Herein, we will test the impact of active rTMS 1 Hz stimulation delivered to the left TPJ on AVH severity scores and use such outcomes to quantify interindividual clinical response variability and test the predictive and explanatory value of a series of magnetic resonance imaging (MRI)-based functional and structural biomarkers assessing interregional interactions. A large battery of clinical, neuropsychological, and motor excitability neurophysiological assessments as well as molecular biology serum assays will be performed in parallel to better understand the determinants of AVH in schizophrenia and pinpoint rTMS therapeutic mechanisms. We hypothesize that rTMS treatment for AVH will show large interindividual response variability, providing a unique opportunity to identify MRI-based structural and functional connectivity biomarkers of positive clinical response to focal neurostimulation.

## Aims and hypotheses

The main objective of this trial is to identify whether the clinical response to rTMS for AVH in schizophrenia patients could be driven by distinct brain connectivity patterns. The clinical response to rTMS is defined as a decrease of 50% or more in the Hallucinations Change Score (HCS) at the end of the active rTMS stimulation (week 3 (W3)) from the baseline (W0) value. We sought to determine whether structural (arcuate fasciculus and corpus callosum) and functional (frontal-temporo-parietal network and the DMN) connectivity differences in AVH-related networks may influence the therapeutic effect of rTMS. These differences may explain why some patients do not respond to active rTMS treatment while others do. We hypothesize that structural and functional connectivity in AVH-related networks would be distinct between schizophrenia patients who respond to rTMS and those who do not.

In a second aim, given rTMS can modulate the resting-state functional connectivity between frontal and temporo-parietal cortices in schizophrenia related to improvements in AVH [9, 24], we sought to evaluate whether there are functional connectivity changes in schizophrenia patients associated with clinical improvement following the rTMS treatment. We hypothesized that functional connectivity changes after rTMS treatment will be only observed in schizophrenia patients who respond clinically to active rTMS (with a reduction of AVH), but not in those who do not.

## Methods/design

### Study design

**Fig. 1:**
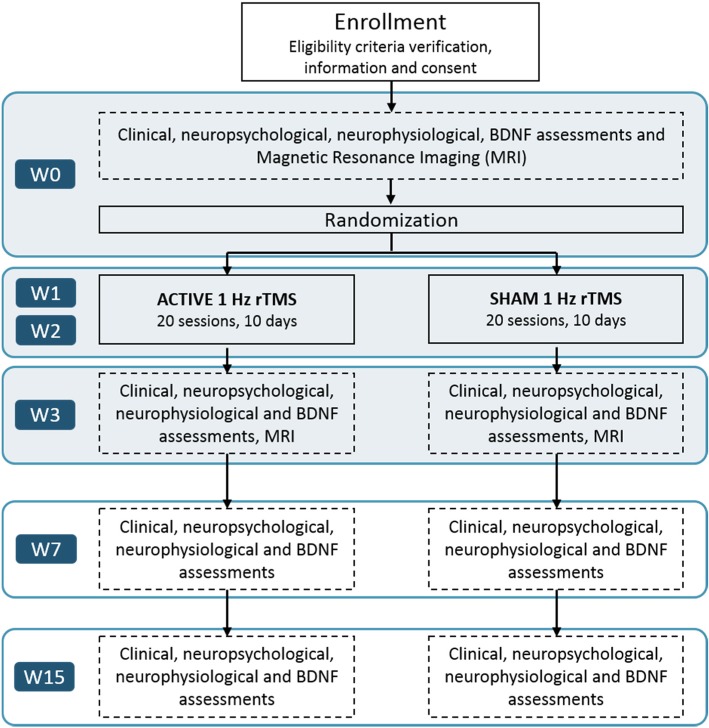
Flow chart of the study design

**Fig. 2:**
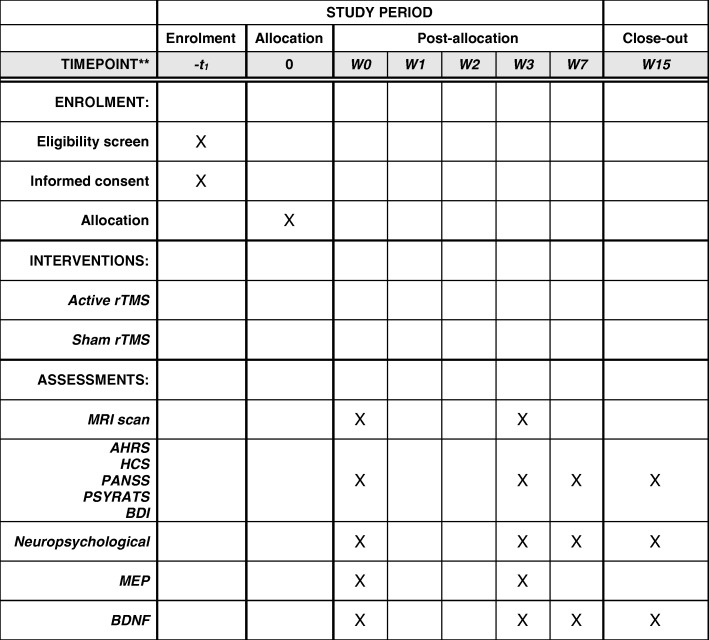
Description of the enrolment, treatment, and assessments during the study protocol *AHRS* Auditory Hallucinations Rating Scale, *BDI* Beck Depression Inventory, *BDNF* brain-derived neurotrophic factor, *HCS* Hallucination Change Score, *MRI* magnetic resonance imaging, *PANSS* Positive And Negative Syndrome Scale, *MEP* motor-evoked potentials, *PSYRATS* Psychotic Symptom Rating Scale, *rTMS* repetitive transcranial magnetic stimulation, *t* time point, *W* week.

This is a randomized, sham-controlled double-blind, monocentric study. Patients will be randomly assigned to one of two arms, either active rTMS or sham rTMS applied to the left TPJ of each patient. Twenty sessions of rTMS treatment will be administered daily (from Monday to Friday, weekends off) at a frequency of 1 Hz for a period of 2 weeks. Clinical, neuropsychological, neurophysiological, and biological evaluations will be carried out before regime onset (W0), on the week following the end of rTMS treatment (W3), and at 1 (W7) and 3 months (W15) after the termination of rTMS treatment. MRI sequences will be recorded at W0 and W3. The first MRI acquisition will be recorded on the Friday of W0 (3 days before rTMS treatment) and the second on the Monday of W3 (3 days after rTMS treatment). Figures 1 and 2 show the experimental design. The protocol study follows the SPIRIT recommendations. For the SPIRIT checklist see Additional file 1.

### Study setting

This trial will be conducted by the Unité de Recherche Clinique of the Etablissement Public de Santé Ville-Evrard (Neuilly-sur-Marne, France). All assessments and rTMS interventions will be performed at the Unité de Recherche Clinique of the Établissement Public de Santé Ville-Evrard. The MRI acquisitions will be performed at the Center for Neuroimaging Research at the Institut du Cerveau et de la Moelle Epinière (Paris, France).

### Participants

#### Recruitment

Participants will be enrolled through psychiatric services of the Établissement Public de Santé Ville-Evrard, hospitals of the region Ile-de-France (within and around Paris, France) and referral by medical practitioners or therapists. The choice of rTMS treatment for schizophrenia patients will be decided by their clinician consultant. Following verification of the eligibility criteria by researchers, patients wishing to participate in the protocol will be included in the study.

#### Inclusion criteria

Patients will have to be diagnosed with schizophrenia according to DSM-5 (Diagnostic and Statistical Manual of Mental Disorders, 5th edition) criteria, suffer from medication-resistant AVH, demonstrate clinical stability for at least 3 months, be aged between 18 and 65 years old, and be right-handed. Medication refractoriness is defined as a failure of treatment with two different antipsychotic drugs, of which at least one is considered atypical. Patients and their physicians will be strongly instructed not to modify the pharmacological treatment right before or throughout participation in the study. Additionally, patients will have to have sufficient knowledge of the French language. All participants of the study will be asked to sign an informed consent form.

#### Non-inclusion criteria

Patients presenting with at least one of the following criteria are not to be enrolled in the study: (1) major psychiatric disorders other than schizophrenia according to DSM-5 criteria; (2) indulging in an addiction (alcohol, psychoactive substances) over the last 12 months; (3) having received rTMS treatment in the last 12 months; (4) participating in a concurrent research protocol; (5) a history of seizures; (6) having any contraindication for rTMS (patient with epilepsy, brain surgery and/or head trauma in the past, use of cardiac pacemaker, or surgical staples on the scalp); (7) having potential contraindications to MRI, e.g. pregnancy or lactating (a negative pregnancy test will be required if the patient is a female in reproductive years who does not use contraception), use of a cardiac pacemaker or surgical staples, patients suffering a neurological disorder, head trauma or claustrophobia; (8) patients with severe cardiovascular disease; (9) patients with medication which could decrease the epileptic threshold (bupropion, methadone, and theophylline); and (10) patients placed in psychiatric care either by the state or a third party.

#### Exit criteria

Patients will be withdrawn from the trial if (1) they simply wish to stop participation; (2) they could not undergo the first MRI session; (3) they did not complete the rTMS sessions; and (4) if they suffered from worsening symptoms.

### Randomization and blinding

The study plans to include a total of 45 patients with schizophrenia randomized into two groups, namely an active rTMS group comprising 35 patients (78% of the total sample) and a sham rTMS group of 10 patients (22%). We will use computer generated blocked-randomization with stratification on gender to get an equal number of males and females on each group. The randomization will be carried out in five blocks of nine participants. Each block will consist of seven patients in the active rTMS group and two patients in the sham rTMS group. Each patient will be randomized during the baseline period by VM. In order to eliminate measurement bias, no member of the trial will be aware of the group in which each patient is randomized to, with the exception of the caregiver performing the rTMS treatment sessions.

At the end of the rTMS treatment (W3), the medical evaluator will debrief with patients on their participation in the protocol and document if they felt they had been assigned to and therefore received an active or sham rTMS treatment.

### Interventions

#### Active rTMS protocol

**Fig. 3:**
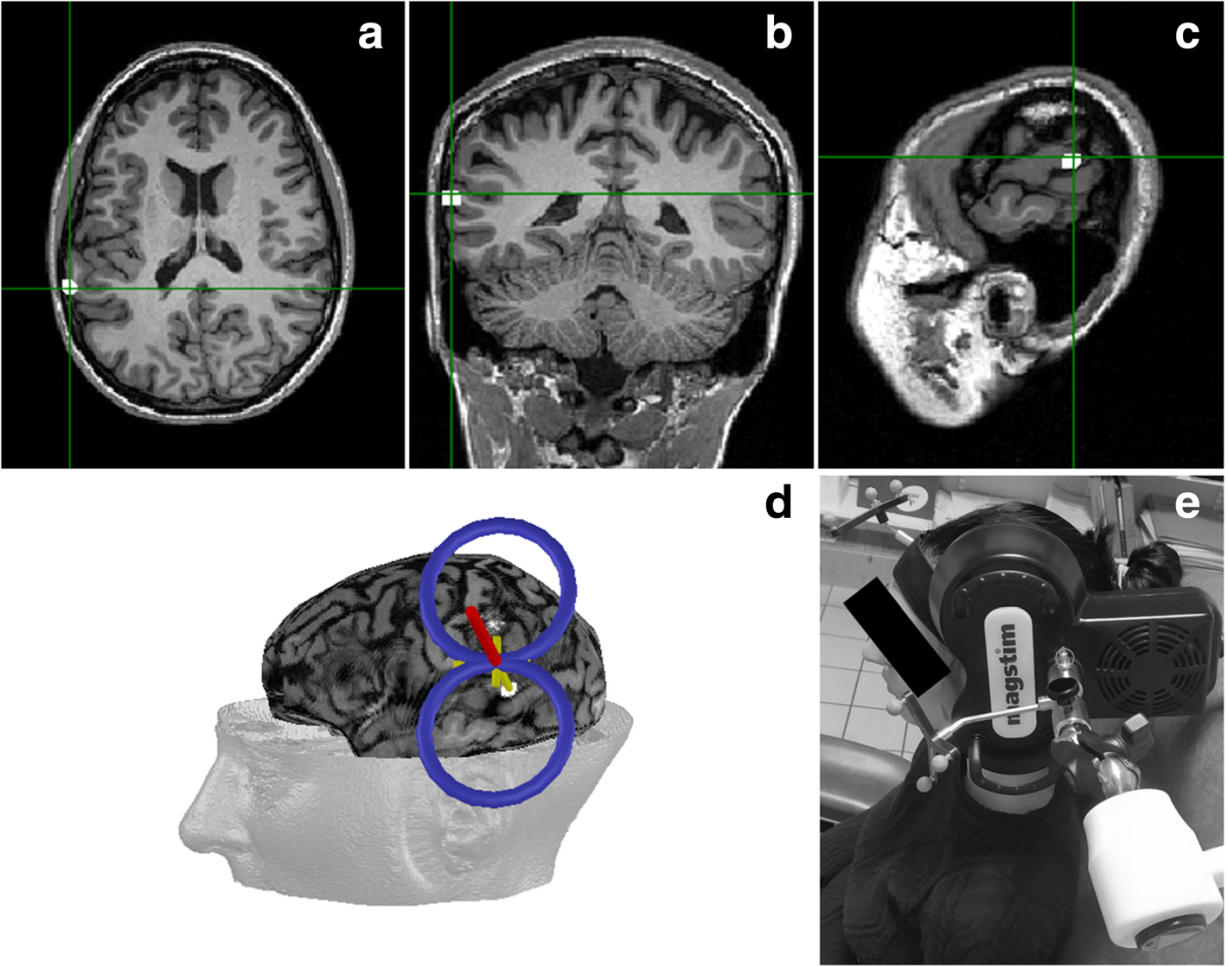
TMS-targeted region and coil placement. The left TPJ will be targeted using established spatial coordinates for the Wernicke’s area in the left TPJ (Montreal Neurological Institute (MNI) coordinates: x = − 69, y = − 41, z = 11; Hoffman et al. [25]). Axial (a), coronal (b), and sagittal (c) MRI views of the location for the TMS targeted left TPJ are shown in this figure. This brain area will be labeled on a 3D rendering of the patient’s individual T1 MRI sequence on each patient. The TMS coil will be positioned tangential to the scalp location overlying the left TPJ using an MRI-based frameless stereotactic neuronavigation system (d and e)

Sessions of rTMS treatment will be carried out with a standard 70 mm figure-of-eight coil (Active Air Film coil, Magstim, Wales, UK), attached to a Magstim Rapid^2^ Stimulator (Magstim, Wales, UK). Participants will be randomly allocated to either the active rTMS (*n* = 35 patients) or to the sham rTMS (*n* = 10) groups. For both groups, the treatment will be administered in two consecutive sessions of rTMS, each delivered for 20 min and interleaved by a stimulation-free 1 h interval. All patients will receive twice-daily rTMS sessions over a period of 2 weeks (from Monday to Friday, weekends off), and therefore accrue a total of 20 rTMS stimulation sessions. The rTMS parameters that will be used are a frequency of 1 Hz (1 pulse per second), an intensity of 100% of the motor threshold (MT), and 1200 continuous pulses per session (thus 2400 pulses per day and 24,000 pulses in total for the whole treatment). The MT is defined as the minimal TMS intensity required to elicit motor evoked potentials (MEPs) measured with electromyographic (EMG) surface electrodes placed on the first dorsal interosseous hand muscle, of at least 50 μV peak-to-peak amplitude, at rest, in 5 out of 10 consecutive trials. The rTMS coil will be applied over the left TPJ, which will be targeted using established spatial coordinates for the Wernicke’s area in the left TPJ (Montreal Neurological Institute (MNI) coordinates: x = − 69, y = − 41, z = 11) [25]. This brain area will be labeled on a 3D rendering of the patient’s individual T1 MRI sequence on each patient. The TMS coil will be positioned tangential to the scalp location overlying the left TPJ using an MRI-based frameless stereotactic neuronavigation system (Brainsight, Rogue Research Inc., Montreal, Canada) (Fig. 3).

#### Sham rTMS protocol

The sham TMS treatment condition will be strictly identical to the active treatment condition thanks to the use of a sham TMS coil (Sham Air Film coil, Magstim, Wales, UK). The sham rTMS coil is indistinguishable in terms of general appearance and shape from an active coil, and able to emulate periodic noise and scalp tactile sensations similar to those produced during the active TMS session for rTMS naïve patients or even experienced healthy subjects [26]. Moreover, this coil has a magnetic shield that reduces the diffusion of the magnetic field under its surface, stimulating only scalp skin receptors or superficial muscles, without inducing any significant physiological transcranial effect on the brain. The two rTMS conditions will be performed in parallel in separate subsets of patients; therefore, patients and evaluators will not be able to differentiate between active and sham stimulation.

### Imaging acquisition

Before and after the TMS therapy, MRI scans will be obtained in all participants on a 3 Tesla scanner (Siemens Healthcare, Erlangen, Germany). To characterize brain structure and navigate the rTMS coil location, we will record three-dimensional, high-resolution, isovoxel, T1-weighted brain volumes acquired with the following parameters: 256 × 240 × 176 matrix size with 176 contiguous slices, field of view (FOV) = 256 mm, 1 mm isotropic resolution, sagittal slice orientation, repetition time (TR) = 2300 ms, and echo time (TE) = 2.98 ms.

To characterize the structural connectivity, diffusion-weighted images (DWIs) will be obtained using a DWI sequence (104 × 104 × 84 matrix size with 60 contiguous slices, FOV = 208 mm, 2 mm isotropic resolution, transversal slice orientation, flip angle = 90°, TR = 3800 ms, TE = 86 ms). The encoding protocol will include 64 different non-collinear directions (gradient factor b = 1500 s/mm^2^) and one image without diffusion weighting used as the reference volume (b = 0 s/mm^2^, b0 image).

To characterize functional connectivity (resting-state functional MRI or rs-fMRI), we will record T2*-weighted MRI sequence applied with an echo-planar gradient (192 × 192 × 162 matrix size with 45 contiguous slices, FOV = 192 mm, 3 mm isotropic resolution, 20*%* interslice gap, transversal and coronal slice orientation, flip angle = 80°, TR = 2500 ms, TE = 30 ms) when subjects are resting while keeping their eyes closed. Patients are instructed to relax, remain still, and not to fall asleep.

After each MRI scan, we will also debrief with patients to investigate and document whether patients experienced hallucinations during the MRI sessions, and if so, in which sensory modalities and during which MRI sequences those might have occurred.

### Outcome measures

#### Primary outcome measures

Our primary aim is to identify brain connectivity differences between patients ‘responders’ and ‘non-responders’ to rTMS treatment. To cluster patients as ‘responders’ or ‘non-responders’, the clinical response to rTMS will be defined as a decrease of 50% or more in the HCS from baseline (W0) at the end of the active rTMS stimulation (W3). This definition is similar to that employed in previous studies [27, 28]. The HCS is described in the Clinical assessments section in the Secondary outcome measures paragraph and will allow analysis of the dataset in search of biomarkers associated with rTMS response, in particular structural and functional connectivity biomarkers.

To identify structural connectivity biomarkers to rTMS therapeutic response, we will compare DWI datasets, acquired at baseline, between ‘responder’ and ‘non-responder’ patients to rTMS. More precisely, we will consider fractional anisotropy (FA) levels along tracts of interest that will be reconstructed from probabilistic tractography algorithms. FA levels will be obtained at several equidistant points along the tract of interest [29]. This procedure will allow evaluation of the existence of global or focal (close to the stimulation site) white matter integrity alterations between ‘responders’ and ‘non-responders’. Tracts of interest will include the fasciculi that are involved in the language network (connections between TPJ and Broca areas, so called the arcuate fasciculi on each hemisphere, and transcallosal connections between the TPJ or Broca areas), and those that are part of the DMN (connections between the superior parietal cortex and prefrontal areas within each hemisphere; transcallosal connections between superior parietal cortex or prefrontal areas). FA values along each tract will be obtained at baseline (W0) and after rTMS treatment (W3) for ‘responders’ and ‘non-responders’.

To identify functional connectivity biomarkers to rTMS response, we will compare functional volumes, obtained at baseline and at rest, between ‘responders’ and ‘non-responders’. We will consider the connectivity strength between areas of networks of interest. Connectivity strength is the degree of dependence between the time courses of two areas (measured with a Pearson coefficient [30] or using dynamic causal modeling (DCM) [31]). The networks of interest will include the DMN (bilateral anterior cingulate cortex, bilateral prefrontal areas, bilateral superior parietal areas, bilateral precuneus) and the language network (bilateral TPJ, Broca areas). Connectivity strength between areas of these two networks will be obtained at baseline (W0) and after rTMS treatment (W3) in ‘responders’ and ‘non-responders’.

#### Secondary outcome measures

##### Sociodemographic and clinical data

For each participant, sociodemographic information (age, sex, educational level, marital status, main activity) and clinical data (onset of schizophrenia, number of hospitalizations, dosage of antipsychotic medication, drug consumption such as tobacco, alcohol and cannabis, history of neurostimulation treatment) will be registered in the patient’s case report form (CRF). Note that the pharmacological treatment is recorded at baseline for each patient and any change will be recorded in the patient’s CRF by the psychiatrist in charge. The Edinburgh handedness inventory will be used to ensure that all patients are right-handed [32].

##### Clinical assessments

The Auditory Hallucinations Rating Scale (AHRS) is widely used to assess the presence and severity of AVH across seven items investigating frequency, level of reality, loudness, number of voices, length of the content, level of distraction, and distress [27]. The HCS is linked to the AHRS and evaluates the percentage of AVH change [33]. On a scale from 0 to 20, a default score of 10 will be attributed to a narrative description of the patient AVH prior to the rTMS sessions (baseline, W0). For the re-assessment of the AVH following rTMS treatment, scores will be evaluated again, and ascribed lower values (with 0 corresponding to total absence of AVH) if AVH severity decreases, whereas a higher score will be ascribed if AVH severity is worse (with 20 indicating twice the severity of AVH compared to baseline). The scoring system of the HCS allows estimation of a percentage of AVH change after rTMS treatment. A score of 5 or less ascribed by the medical evaluator at W3 indicates a decrease of 50% or more of AVH. In this case, the patient will be considered a ‘responder’. Otherwise, the patient will be considered a ‘non-responder’. The AHRS and HCS will be employed to assess the severity of AVH and the efficacy of rTMS therapy. AHRS and HCS will be recorded at each time point of this study (W0, W3, W7, and W15).

The Positive And Negative Syndrome Scale [34] is a 30-item scale, which is widely used to assess the overall disease severity of schizophrenia such as psychosis-related symptoms (hallucinatory behavior, delusions, emotional withdrawal, stereotyped thinking) and general psychopathology (including ‘poor attention’, depression and anxiety). In addition, the Psychotic Symptom Rating Scale (PSYRATS [35]), a 17-item scale, will be employed to evaluate the psychotic symptoms (hallucinations and delusions) and the Scale for the Assessment of Negative Symptoms [36], a 25-item scale, will be used to assess the negative symptoms in schizophrenia.

Comorbid depressive symptoms will be investigated with the Beck Depression Inventory [37], a 13-item, self-report rating inventory and the Calgary Depression Scale for Schizophrenia, a 9-item structured interview scale [38].

Finally, the Short Form (36) Health Survey, a 36-item scale, will be used to obtain a measure of the health-associated quality of life such as physical functioning, bodily pain, role limitations due to physical health problems, role limitations due to personal or emotional problems, emotional well-being, social functioning, energy/fatigue, and general health perceptions [39].

All of the above clinical scales will be recorded at baseline (W0) and at several stages following the end of the rTMS treatment (W3, W7, and W15). More precisely, clinical assessments at W3 will be mainly performed on Tuesday or Wednesday. It will be used to follow symptomatology of schizophrenia across this study and to investigate the impact of rTMS treatment on such symptoms, in particular AVH. Moreover, baseline AVH severity levels could also constitute a marker of the rTMS response in schizophrenia patients.

##### Neuropsychological assessments

A neuropsychological assessment evaluating performance on several cognitive domains (more specifically, executive function, mental flexibility, inhibition, verbal fluency, memory, and writing abilities) will be carried out on each participant. These data recorded at baseline (W0) will be used to evaluate cognitive impairment and identify potential neuropsychological markers associated with rTMS response. Carried out in the post rTMS period, this assessment will also allow study of the impact of multiday rTMS regime on cognitive function.

The assessment will be performed prior (W0) and, most importantly, at several intervals following the end of the rTMS regime (W3, W7, and W15). It will include the following tasks: the Trail Making Test A and B [40], the Stroop test [41], the Hanoi Tower [42], the Wisconsin Card Sorting Test [40], a Verbal Fluency task [40], the California Verbal Learning test [43], the Digit Span and Visual Memory tests [44], an autobiographical memory task [45], and a dictation writing task [46].

##### Neurophysiological assessments

Before and after rTMS treatment, cortical excitability measures will be assessed for each patient using a standard 70 mm figure-of-eight coil (MCF-B65 Butterfly Coil, MagVenture A/S, Farum, Denmark) attached to a MagPro R30 stimulator with MagOption (Medtronic A/S, Copenhagen, Denmark). The MT will be assessed using the TMS Motor Threshold Assessment Tool (MTAT 2.0, http://www.clinicalresearcher.org/software.htm). Self-adhesive, solid gel-coated disposable Ag*/*AgCl surface electrodes placed on the first dorsal interosseous hand muscle will be employed to record these measures. Peak-to-peak MEP amplitudes will be recorded at 120% of the individual MT.

Moreover, several neuroexcitability abnormalities have been described in schizophrenia patients, essentially in cortical inhibition. A deficit of intracortical inhibition (ICI) is the most robust finding in this patient population, whereas deficits of the cortical silent period (CSP) and MT have been reported inconsistently [47]. In our protocol, the CSP, ICI, and intracortical facilitation (ICF) will also be recorded to monitor potential motor intracortical changes following the rTMS treatment over the left TPJ. To this regard, a recent clinical case reported indirect signs of clinical improvement and motor excitability changes via CSP in a pontine stroke patient after rTMS treatment delivered over the left TPJ to treat their AVH [48]. The CSP will be evoked by applying TMS intensity at 120% of the individual MT while patients will perform a hand isometric contraction of approximately 50% of their maximum force. The duration of the CSP will be measured from the end of MEP until the re-occurrence of EMG activity. ICI and ICF will be determined using a paired-pulse paradigm. The conditioning subthreshold stimulus, which will be set at 80% of MT, will be preceded by a suprathreshold stimulus test at 120% of the MT. Interstimulus intervals will be set at 2 and 4 ms for ICI and at 10 and 15 ms for ICF. The EMG activity will be recorded by a computer using System PLUS EVOLUTION software (version 1.04, Micromed, Mâcon, France).

The MEPs amplitude at 120% of the MT as well as CSP, ICI, and ICF will be obtained prior (W0) and following the rTMS treatment (W3, W7 and W15) and will be used to estimate individual levels of motor cortical excitability between ‘responder’ and ‘non-responder’ patients, monitoring potential changes of such across treatment and during follow-up. Electrophysiological measures comparing pre versus post rTMS between ‘responders’ and ‘non-responders’ could constitute a potential explanatory marker informing on rTMS mechanism of action.

##### Biological measures from blood samples

Brain-derived neurotrophic factor (BDNF) is a neurotrophin that plays a key role in neuronal survival and synaptic plasticity (i.e. morphological and physiological changes of synapses induced by neuronal activity changes). BDNF levels may reflect a decrease or increase of brain activity and provide a potential explanatory marker of rTMS effects [49]. Serum BDNF levels will be measured and compared between ‘responder’ versus ‘non-responder’ patient groups and also between active rTMS versus sham rTMS stimulated groups of patients. This will allow investigation of rTMS-induced brain plasticity changes [50].

To this end, blood samples will be collected from all patients in the morning (before 10:00 a.m.) at baseline (W0) and after the end of the regime (W3, W7, and W15) in a 3.5 mL serum separator tube (SST™ II Advance, BD Vacutainer^®^, New Jersey, USA). Blood samples will be stored at room temperature for 20 min, then centrifuged at 3500 rpm at 4 °C for 20 min. Serum will be separated from blood and kept in a refrigerator at − 30 °C prior to analyses. Serum optical density will be measured at 450 nm using a microplate reader (EZ Read 400, Biochrom, Cambridge, UK). BDNF concentrations will be determined by comparing the optical density to a standard curve.

### Participant timeline

The week prior to the onset of the 10-day rTMS treatment, clinical scales, neuropsychological tests, structural and functional MRI recordings, neurophysiological motor excitability evaluations, and biological markers from blood serum samples will be obtained. Once baseline recordings are completed, the rTMS treatment will start. Each patient will receive a total of 20 sessions of 1 Hz rTMS (1200 pulses/session, two sessions/day from Monday to Friday, over two consecutive weeks), delivered on the left TPJ region. Once the rTMS treatment is completed, many of the same clinical, neuropsychological, neuroimaging, and neurophysiological measures determined during the baseline prior to rTMS regime onset will be recorded again to evaluate the impact of stimulation (Fig. 1).

### Data collection and management

A CRF will be used to collect the data for each participant. To preserve patient anonymity, an identification code is allocated during the enrollment phase allowing patient identification on the CRF. Throughout this trial, CRFs will be reviewed to ensure complete and accurate collection of data following each assessment. CRFs will be stored in a locked cupboard for data security. MRI data will be stored on a computer secured by a password and will be identified with the patient’s identification code to preserve anonymity.

### Statistical analysis

#### Sample size

The aim of this trial is to investigate the difference in brain functional and structural connectivity in schizophrenia patients receiving an active rTMS treatment and clinically responding to it or not as assessed by an amelioration of their AVH symptoms. Therefore, the sample size was calculated to determine the number of patients to be included in the active rTMS group on the basis of the primary outcome, namely the difference in FA values at baseline between ‘responders’ and ‘non-responders’. Unfortunately, no study provides a significant difference of rs-fMRI measures or FA values for a given tract (such as the left arcuate fasciculus) before and following a clinically effective versus a non-clinically effective rTMS treatment in schizophrenia patients, which would allow us to accurately estimate the adequate sample size for our trial. Nonetheless, FA values are highly reproducible and have a reliable estimation of variance. Therefore, we focused on FA values to determine sample size. Therefore, on the basis of a significant difference in the FA values between schizophrenia patients and healthy subjects for the left arcuate fasciculus [51] and assuming an α risk of 0.05 and a 1-β power of 0.80, we estimated the need to include 32 patients for the active rTMS group. To overcome an estimated 10% drop-out, we increased our recruitment needs for active rTMS group to 35 patients. Given a proportion of ‘responders’ and ‘non-responders’ to rTMS treatment as the one we propose for AVH in schizophrenia estimated at 40% and 60%, respectively [52], we will divide our cohort into 14 ‘responders’ and 21 ‘non-responder’ patients. According to a prior study [22], a minimum of 14 participants per group (‘responders’ or ‘non-responders’) has been deemed sufficient to conduct statistical analysis in neuroimaging and detect a significant effect of rTMS on brain structural connectivity. Moreover, a sham rTMS group of 10 patients will be followed in parallel to control for the placebo rTMS effect on AVH. As a result, the total number of participants required for the study is of 45 patients.

#### Data analysis

In order to meet the objectives of this trial, structural and functional connectivity data will be processed in two different ways. First, we will compare measures, obtained at baseline (W0), between ‘responder’ and ‘non-responder’ patients submitted to active rTMS to identify potential brain biomarkers associated with rTMS response. Secondly, we will compare these same data, before versus after active rTMS, between groups of ‘responders’ and ‘non-responders’ to determine explanatory markers of active rTMS-induced AVH improvement. For both steps, we sought to show a significant difference between ‘responders’ and ‘non-responders’. Thirdly, additional exploratory analysis will be performed on clinical, neurophysiological, neuropsychological, and biological measures.

#### MRI data analysis

##### Regions of interest (ROIs)

Two spherical ROIs corresponding to the rTMS target (left TPJ) and its homotopic region in the right hemisphere (right TPJ) will be generated using the MarsBaR toolbox (http://marsbar.sourceforge.net/) [53]. Each sphere of a 5 mm radius will be centered at x = − 55, y = − 41, and y = 11 for the left TPJ, and at x = 55, y = − 41, and y = 11 for the right TPJ. MNI atlases will be used to define ROIs containing the nodes of the DMN and the language network. The masks of the left and right frontal inferior gyri and posterior cingulate cortex will be extracted from the WFU PickAtlas (http://fmri.wfubmc.edu/software/PickAtlas). A mask of the corpus callosum will be manually drawn on a T1-weighted MNI template using the Functional Magnetic Resonance Imaging of the Brain (FMRIB) Software Library (FSL 5.0.9, http://www.fmrib.ox.ac.uk/fsl/). For tractography analyses, the same ROI will be de-normalized from the MNI space to the individual space using the inverse transformation obtained from the VBM8 toolbox.

##### Structural connectivity analysis

Diffusion images will be preprocessed using FSL software and then processed for probabilistic diffusion tractography with MRtrix3 software (http://www.mrtrix.org/). Diffusion volumes will be corrected for motion and geometric distortions induced by eddy currents. The constrained spherical deconvolution method will be used to estimate the fiber orientation distribution function (ODF) in MRtrix3 [54]. In the native space of each subject, a seed-to-target analysis will be performed to reconstruct the tracts of interest included in the DMN and the language network (arcuate fasciculus, corpus callosum).

Fiber-tracking maps will be created for each subject by using FSL software (data preprocessing) and MRtrix3 software (diffusion images) [54]. Using a voxel-wise model of diffusion (the Q-ball model), the maximum-likelihood solution for fiber orientation within each voxel will be represented by an ODF on the location of the fiber trajectory. The ODF characterizes the orientation dependency of the diffusion probability density function of water molecules in several possible directions for each voxel. This model can be used to track complex fiber configurations. The ODF information obtained from constrained spherical deconvolution will be used with a suitable fiber-tracking algorithm to infer connectivity of crossing fibers. We will use a probabilistic streamline algorithm with the entire ODF as a probability density function (ODF threshold = 0.1; step size = 0.5 mm x voxelsize; radius of curvature = 1 mm; up-sampling of DWIs, data to 1 mm). In the native individual space, we will perform a seed-to-target analysis from ROIs of the networks of interest (see Region of interest section). These regions will include the bilateral TPJ, the inferior frontal gyri and the corpus callosum. We will use the following probabilistic tractography algorithm: the number of fibers (streamlines) connecting a seed voxel to a target voxel will be calculated by sampling 1 million draws for each fiber connecting the seed to the target. The pair-wise connections of the DMN and the language networks will be reconstructed for each subject.

Once tracts are reconstructed, one complementary measure will be considered. FA values will be measured along the tracts of interest. Along-tract measures of FA will be obtained on the basis of B-spline resampling of the fibers and averaging the FA values for each individual fiber on a given location (elastic model with 30 points in space at analogous anatomical locations in each individual) [29]. For instance, for the language network, the posterior location will be defined on the ROI of the TPJ, whereas the anterior location will be defined as the level of the ROI of the Broca areas. Mean FA values will be calculated at each point of the mean fiber along the *y* axis to check for local differences at specific points of the tract. A linear mixed-effects model will be applied serially for each tract and permutation methods will be performed to adjust the *p* values and control the Type 1 error.

##### Resting-state functional connectivity analysis

Statistical parametric mapping software (SPM8, Wellcome Department of Cognitive Neurology, London, UK) will be used for image processing (http://www.fil.ion.ucl.ac.uk/spm/). The functional images will be interpolated in time to correct for phase advance during volume acquisition, and will be realigned with the first image of each session. The anatomical image and the realigned functional images of each subject will then be normalized to a common standard space by using the MNI template (DARTEL [55]). The functional data will be spatially smoothed with a 4-mm full-width at half-maximum Gaussian filter and temporally filtered (0.01–0.08 Hz) with a 128-s period high-pass filter.

Functional connectivity analysis will be performed [56–59]. The correlations in spontaneous BOLD fluctuations reflect the inter-regional correlations in neuronal variability [60, 61]. The averaged time course will be obtained from a previously defined ROI (posterior cingulate cortex for the DMN, TPJ for the language network). The correlation analysis will be performed in a voxel-wise way to generate the functional connectivity map. The correlation coefficient map will be converted into a *z* map by Fisher’s *r*-to-*z* transform to improve the normality [62] (Data Processing Assistant for Resting-State fMRI [63], http://www.restfmri.net). For each network of interest (DMN, language network), the second level analysis will be performed using a two-tailed, two-sample *t* test on the *z*-score maps to show group differences on connections of the DMN and language networks.

Effective connectivity analysis will be performed using DCM with the DCM10 routine implemented in Statistical Parametric Mapping software 12. The first eigenvectors will be extracted from the ROIs representing the DMN and language networks (see ‘Regions of interest’ section). The a priori models will be based on anatomical connections, considering a fully connected model of the DMN and language network. Intrinsic connectivity will be defined as the endogenous connectivity parameter without driving input [31]. The intrinsic connectivity values will be obtained for each subject in the best model of each network that will be considered for group analysis. Bayesian model selection [64, 65] will be used to determine the best model between unilateral and bilateral connections. Expected posterior model probabilities and exceedance probabilities will be computed. The group analysis on the DCM parameters will only include the model that will best fit the data. Within each group, one sample *t* test will be conducted to examine whether the parameters of the model that best fits the data will have significant non-zero values. Two-sample *t* tests will be used to identify group differences in intrinsic connectivity on each connection. The same will be performed for the DMN to examine whether rTMS has a specific effect on the language network or a more global effect on attentional mechanisms.

##### Demographic, clinical, neurophysiological, neuropsychological and biological data analysis

Socio-demographic data will be compared between the active rTMS and the sham rTMS groups, and also between the ‘responder’ and ‘non-responder’ groups, using a two-sample *t* test (Student’s *t* test) or a χ^2^ test.

To control for the rTMS treatment efficacy, a repeated-measures analysis of variance (ANOVA) will be performed on AHRS with time (before/after rTMS) as the within-subject factor and group (active rTMS/sham rTMS) as the between-group factor (based on the per-protocol principles). The same strategy will be followed for clinical, neurophysiological, neuropsychological, and biological data. Post-hoc pair-wise comparisons will be performed using a two-sample *t* test (Student’s *t* test) or χ^2^ test. Bonferroni correction will be applied as a multiple comparison adjustment to reduce the chances of a false positive result. Normality of data distribution (Shapiro–Wilk’s test) and equality of variances (Levene’s test) will be previously verified. All data will be presented as mean ± standard deviation. For all tests, statistical significance will be set as *p* ≤ 0.05.

## Discussion

Several meta-analyses support significant reduction of AVH severity in schizophrenia following a multi-day 1 Hz rTMS regime [12–19], spurring hopes for rTMS for this indication. Nonetheless, the high level of interindividual variability in clinical responses has weakened enthusiasm about its clinical efficacy and the topic remains debated. In this context, the identification of biomarkers predictive of positive clinical response could help clarify the situation and provide more effective, suitable, and customized treatments for AVH in schizophrenia. Moreover, by identifying patients who are most likely to respond before therapy is applied, clinical indications could be fine-tuned and, thus, general outcomes could be improved. In our trial, functional and structural neuroimaging datasets recorded prior and following the rTMS therapy will be employed to better characterize mechanisms underlying the emergence of AVH, evaluate the impact of a multiday rTMS regime, and gauge ability to drive enduring recovery.

The protocol proposed for our trial follows previously established recommendations on therapeutic uses of rTMS for the treatment of AVH, using 1 Hz rTMS over the left TPJ region [66]. However, to date, no clear guidelines exist to titrate other stimulation parameters, such as rTMS intensity (established between 90% and 100% of the MT) or the number of total sessions (or cumulated sessions per day) necessary to achieve stable recovery in the shortest possible period (10 sessions over 1–2 weeks remain the most common protocol), which vary greatly across studies [66]. Moreover, no established guidelines seem to exist yet with regards to the maximal number of pulses to be delivered per session. Nonetheless, to our knowledge, no study included in the guidelines for therapeutic use of rTMS [66] or cited in the 11 meta-analyses addressing the effect of rTMS on auditory hallucinations in schizophrenia from 2007 to 2018 [13–18, 67–69] seem to use more than 1200 pulses per session. To remain consistent with prior literature and convince the Institutional Review Board of the safety of our interventions we took the decision to align ourselves with established recommendations or prior peer-reviewed publications in the field.

In this protocol, we chose to administer two sessions of 1200 pulses of 1 Hz rTMS per day (interleaved by a TMS-free 1 h interval) across two consecutive weeks, from Monday to Friday (10 days in total). The doubling of daily rTMS sessions aims to increase the total number of delivered stimuli, enhance effect magnitude and duration, and shorten stimulation regime duration. To this regard, fundamental studies evaluating changes of primary motor cortex excitability in healthy participants have shown, for example, that the higher the number of stimuli, the longer the duration of rTMS inhibitory effects [70, 71].

During the description of the study to patients, the investigators will clearly specify that rTMS therapy occurs daily over two consecutive weeks (except on weekends). After the inclusion phase, a prospective schedule for the rTMS sessions (and other appointments) will be proposed to be approved by each patient. If necessary, the appointment time can be modified and adapted to the patient requirements. If once duly informed about the schedule, a patient declares that they cannot attend all the planned rTMS sessions over 2 weeks, they will not be included in the study. In that case, an active rTMS treatment with a more flexible schedule can be offered to these patients, outside this research protocol. If the participant misses an appointment, it is possible to shift the sessions of a day. In case a patient stops attending the rTMS sessions before the end of the treatment or if they are unable to attend to more than two consecutive rTMS sessions for any reason, investigators will consider that the established research protocol is no longer respected. The participant will then be withdrawn from the study and their data will not be considered in the analyses. This situation will not suppose any change for the medical attention or treatment the patient undergoes in our institution.

Regarding potential rTMS targets in AVH, work published by Hoffman et al. [25, 72] has shown that active (but not sham) rTMS over the left TPJ induces higher levels of clinical improvement than stimulation on temporal and frontal areas. Therefore, the current protocol will employ MRI-based neuronavigation to localize the Wernicke’s area in the left TPJ according to specific spatial coordinates (MNI coordinates: x = − 69, y = − 41, z = 11) on each individual participant MRI.

It is also important to mention that, although the main and primary outcome goal of our project is the identification of structural and functional brain biomarkers of clinical response, our rTMS protocol includes a sham rTMS group to control for the placebo effect of rTMS therapy on AVH. In an attempt to meet the main objective of our trial within a reasonable time period, which is to identify biomarkers associated with clinical response, we here decided to assign as many participants as we could possibly recruit and treat (given sample size calculations and allotted time and resources) to an active rTMS treatment. Consequently, an unequal lower number of patients (*n* = 10 patients, i.e., 22% of the final sample) will be randomized to the sham rTMS group which is used to verify the known lack of recovery of enduring AVH when no effective intervention is applied.

In our study, we propose a stratified randomization based on sex only although a stratification by sex and age would be more complete. Nonetheless, we are bound to study two groups (active rTMS *n* = 35 and placebo rTMS *n* = 10) with an unequal number of patients, and therefore the stratification on sex as well as age ranges would be more complex. We have currently defined two strata – male and female. If we were to add the variable ‘age’, we would have to deal with a much higher number of strata (two for gender and, for example, three for age: 18–35, 35–50, and 50–65 years old), and this would significantly slow down or seriously compromise the recruitment process in the time allotted to complete the study. Nonetheless, sex and age will be added as covariables in the statistical analyses.

Importantly, in our trial, we will define clinical response to active rTMS as a reduction of 50% in the HCS measured at the end of rTMS treatment (W3) compared to baseline evaluation (W0); this criteria of response is commonly found in rTMS studies in schizophrenia [27, 52, 73, 74]. Nevertheless, the response criteria may vary in some studies in which ‘responders’ are defined as patients showing a reduction of 30% in the PSYRATS [75, 76] or the AHRS scale [77]. Even though different scales such as the PSYRATS or the AHRS scales have both been shown to exhibit good psychometric reliability, the HCS seems to be the most sensitive to rTMS effects on AVH [13, 69] and thus will be applied to classify patients as ‘responders’ or ‘non-responders’ to rTMS. Using this same criterion to classify patients with positive (for ‘responders’) or negative (for ‘non-responders’) clinical responses to rTMS, our protocol predicts 40–50% success rate, thus proving beneficial for at least half of actively stimulated patients.

With regards to the neuroimaging datasets, three sequences (structural 3D-T1 MRI, functional rs-fMRI, and DWI) will be acquired prior and following the multiday rTMS protocol. MRI acquisition sessions will take approximately 30 min for each patient. Schizophrenia patients could have difficulty staying quiet and motionless for a long time in an unnatural environment such as the MRI bore. Thus, the final rs-fMRI sequence chosen, which is relatively short compared to similar studies in healthy participants, is the result of a tight compromise that aims to optimize the investigation of brain functional connectivity in this specific population.

In our trial, we will also determine the levels of BDNF in blood serum for each subject at baseline (W0) and at several stages following the end of the rTMS regime (W3, W7 and W15). BDNF plays a key role in neuronal survival, neurogenesis, the growth of dendrites and axons, synapse formation and synaptic strength gain [78]. Thus, increases of this factor may likely reflect or impact the level of brain plasticity and excitability changes induced by rTMS [49], which could potentially correlate with the magnitude of rTMS effect on AVH in schizophrenia patients. Moreover, baseline blood levels of BDNF could be predictive of clinical response.

Our trial also integrates an evaluation of several cognitive domains, which can either be altered in schizophrenia patients or could be modified by the 10-day rTMS treatment delivered to the left TPJ, thus informing about the cognitive domain selectivity of stimulation. The completion of all these tests takes at least 2 h at baseline (W0) and post rTMS regime (W3) evaluations. For practical reasons and to ensure patient compliance and reliability, evaluations have been planned and adapted to last no more than 1 h. Consequently, several tests (particularly the Hanoi Tower, the verbal memory task, the task of Corsi or MEM battery, and an autobiographical memory task) have been removed. For the same reason, detailed neurophysiological measurements will not be performed at W7 and W15 post rTMS treatment.

Another point we discussed about neuropsychological assessment is the estimation of a patient’s premorbid intellectual functioning. A very detailed assessment of premorbid intellectual status was not included in our study since it would increase the time spent on cognitive assessments for patients. Indeed, this assessment takes approximately 90 min using the Wechsler Adult Intelligence Scale or 40 min using a short form of this. An alternative, the National Adult Reading Test, is used for estimating premorbid intelligence levels and could take just a few minutes. Nonetheless, this test consists of 50 words, graded in difficulty, whose pronunciation cannot be determined from their spelling. Unfortunately, our protocol will include patients with sufficient knowledge of the French language although not necessarily be French-native speakers and, as such, the outcomes of the National Adult Reading Test could be biased.

In conclusion, the current clinical trial will investigate how brain structural and functional connectivity at the network level may influence rTMS impact on AVH severity, and also address how the rTMS treatment can modulate brain function and induce clinical improvement for this symptom in schizophrenia patients. Our study will provide new insights to treat AVH with rTMS in schizophrenia and, by identifying structural and functional connectivity biomarkers of the clinical response to rTMS stimulation in AVH, eventually contribute significantly to refine this indication and allow the customization of therapeutic protocols.

### Trial status

The first version of this protocol was approved on 28 April, 2015. The first participant was included in the study on 14 November, 2015. The local Ethics Committee accepted an amendment we requested to add a sham rTMS group on 1 December, 2015. This second and final version of the protocol is currently in the recruitment phase. The end of the recruitment phase is currently estimated for December 2018.

## Additional file

Additional file 1: SPIRIT 2013 Checklist: recommended items to address in a clinical trial protocol and related documents. (DOC 122 kb)

## Abbreviations

AHRS: Auditory Hallucinations Rating Scale
AVH: auditory verbal hallucinations
BDNF: brain-derived neurotrophic factor
CRF: case report form
CSP: cortical silent period
DCM: dynamic causal modelling
DMN: Default Mode Network
DWI: diffusion weighted-images
EMG: electromyographic
FA: fractional anisotropy
FOV: field of view
FSL: Functional Magnetic Resonance Imaging of the Brain (FMRIB) Software Library
HCS: Hallucinations Change Score
ICF: intracortical facilitation
ICI: intracortical inhibition
MEPs: motor-evoked potentials
MNI: Montreal Neurological Institute
MRI: magnetic resonance imaging
MT: motor threshold
ODF: orientation distribution function
PSYRATS: Psychotic Symptom Rating Scale
ROI: region of interest
rs-fMRI: resting-state functional MRI
rTMS: repetitive transcranial magnetic stimulation
TE: echo time
TPJ: temporo-parietal junction
TR: repetition time
W: week
